# Hooding cobras can get ahead of other snakes in the ability to evoke human fear

**DOI:** 10.1007/s00114-024-01952-2

**Published:** 2024-12-04

**Authors:** Daniel Frynta, Iveta Štolhoferová, Hassan Sh Abdirahman Elmi, Markéta Janovcová, Veronika Rudolfová, Kateřina Rexová, David Sommer, David Král, Daniel Alex Berti, Eva Landová, Petra Frýdlová

**Affiliations:** 1 Department of Zoology, Faculty of Science, Charles University, Prague, Czech Republic; 2 Department of Biology, Faculty of Education, Amoud University, Borama, Somaliland

**Keywords:** Fear, Evolutionary psychology, Cross-cultural comparison, Hooding, Ophidiophobia, Specific phobias

## Abstract

Fear of snakes is common not only in humans but also in other primates. Consequently, snakes are salient stimuli associated with prioritized attention, early detection and emotional significance. This has been interpreted as an adaptive evolutionary response of the primate brain to a risk of envenoming by a hidden snake. However, the struggle between mammals and snakes is not one-sided. Humans and carnivores regularly kill snakes, and thus snakes develop deterring defensive behaviour that may directly evoke enhanced fear. Here, we show that snakes depicted in threatening posture evoked on average more fear than those in resting posture. Significantly, African (Somali) and European (Czech) respondents considerably agreed on the relative fear elicited by various snakes. Nonetheless, not all defensive postures are equally efficient. Threatening cobras were perceived as top fear-evoking stimuli, even though most of them are not considered very frightening in resting posture. This effect can be attributed to their conspicuous hooding posture which evolved into an efficient warning signal for mammalian predators. Our result demonstrates that cobras are more effective than other snakes in the ability to evoke human fear by a simple behavioural display—hooding. This can be primarily explained by the behavioural evolution of cobras which successfully exploited pre-existing cognitive mechanisms of mammals. Whether human ancestors cohabiting with deadly venomous cobras further improved their fear response to hooding is uncertain, but likely.

**Supplementary Information:**

The online version contains supplementary material available at 10.1007/s00114-024-01952-2.

## Introduction

Snakes belong to the most fear-evoking animal stimuli, and this can be demonstrated cross-culturally (e.g. Europe: Janovcová et al. 2019; Landová et al. 2020; Polák et al. 2020; Staňková et al. 2021, Africa: Onyishi et al. 2021; Frynta et al. 2023a). Also, many other primates exhibit avoidance and/or fear of snakes (e.g. Etting et al. 2014; Weiss et al. 2015). As snakebites still represent a serious risk for both humans (Chippaux 2011; Halilu et al. 2019; Afroz et al. 2024; Fernández and Youssef 2024,) and other primates (Headland and Greene 2011), this fear is relevant and attributable to an evolutionary adaptation of primate brains to this selective pressure (Isbell 2006, 2009; Zsidó et al. 2023). Most snakebites occur when people accidentally step on a snake with an unprotected foot or otherwise provoke a defensive bite, often without realising the snake’s presence until the last moment. Consequently, extensive research has primarily focused on how humans detect hidden snakes in their environment and avoid them, particularly in phenomena such as detection (Öhman et al. 2001; Öhman and Mineka 2001, 2003; LoBue and DeLoache 2011; Soares et al. 2014; Van Strien et al. 2016; Van Strien and Isbell 2017, Coelho et al. 2019, Zsidó et al. 2024b), recognition (Wombolt and Caine 2016) and prioritisation of snake-related stimuli (Jensen and Caine 2021; but see Lazarević et al. 2020; Zsidó et al. 2024a).

The adaptive response of primates to snake-related hazards is thought to be primarily related to increased visual attention and rapid detection of snakes (Gomes et al. 2018) and to unconscious fear processing in the brain, the so-called low road to the amygdala (retina-colliculi superiores-pulvinar-amygdala-automatic motor response; Carli and Farabollini 2022). Most of the research to date utilizes snake images as fear-eliciting stimuli. Subjective evaluation of snake images according to the degree of experienced fear involves both unconscious automatic responses to potential threat and subsequent conscious emotional appraisal and emotion regulation. Interestingly, regions of the low amygdala pathway are activated in response to snake stimuli in tasks involving both implicit (automatic) and explicit (goal-directed and experience-driven) visual and emotional processing of snake stimuli in the human brain (reviewed in Almeida et al. 2015). Unconscious visual processing likely influences conscious emotional appraisal due to high subcortical-cortical connectivity, indicating that both automatic (LeDoux 2012) and conscious emotional and cognitive processes are at play (reviewed in Pessoa and Adolphs 2010; Dinh et al. 2021; see also Nicula 2020). Thus, the subjective evaluation of snake images is influenced by the strength and feedback regulation of consciously and unconsciously processed fear. The relationship between humans or other primates on one side and snakes on the other is certainly not one-sided (e.g. Falótico et al. 2018). While humans and other primates may be threatened by defensive snake bites (McGrew 2015; Perry et al. 2020; Afroz et al. 2024), snakes, in turn, face the threat of being killed by humans (Whitaker and Shine 2000; Secco et al. 2014; Larson et al. 2024). People from various cultures deliberately kill snakes (Yorek 2009, Balakrishnan 2010, but see Ballouard et al. 2013), and also, non-human primates regularly mob and exceptionally also kill snakes (e.g. Galat-Luong 1991; Crofoot 2013). Moreover, there are numerous specialised predators of snakes among birds of prey (e.g. African secretary bird, *Sagittarius serpentarius*) or small carnivores. Some snake enemies, e.g. Egyptian mongoose (*Herpestes ichneumon*) and meerkat (*Suricata suricatta*), possess considerable resistance to snake venoms (Khan et al. 2020). Consequently, both parties strive to avoid undesirable encounters.

It was demonstrated that venomous snakes do not differ from non-venomous ones in their longevity (Hossie et al. 2013). In an evolutionary logic, this provides a piece of indirect evidence that the venom apparatus by itself is not a sufficient protection against predators (Hossie et al. 2013). As a result, snakes have developed a range of other protective mechanisms. Besides hiding and camouflage (Allen et al. 2013; Valkonen et al. 2020; Cox and Davis Rabosky 2023) and passive protection via dermal armour (Frýdlová et al. 2023), defensive behaviour is particularly noteworthy, as it allows the snake to warn and deter potential predators. Let us set aside chemical repellent signals and the feigning of death (thanatosis, e.g. Golubović et al. 2021; Magallón et al. 2021; Pandey et al. 2022) as well as aposematic colouring, which may be visible even when the snake is at rest (Mouy 2024). The defensive strike is a common form of snake defence, but the strikes are not always accompanied by an injection of venom (Hayes et al. 2002; Araújo and Martins 2006, 2007; Piao et al. 2021). A frequent defensive warning is optical signalling mediated by threat postures (Gibbons and Dorcas 2002; Shine et al. 2002) like coiled position, body inflation and/or elevated neck presenting the hood. The posture could be accompanied by stereotyped movements, tail rattling and demonstrative strikes (Allf et al. 2016). Moreover, there is acoustic signalling such as hissing (Kinney et al. 1998; Aubret and Mangin 2014), scale rubbing (e.g. in the carpet vipers of the genus *Echis*, Pitman 1972; and egg-eating snakes of the genus *Dasypeltis*, Gans and Richmond 1957), growling of king cobras (*Ophiophagus*, Young 1991) and tail rattling in rattlesnakes (*Crotalus* and *Sistrurus,* Fenton and Licht 1990; Cook et al. 1994). The defensive behaviour of rattlesnakes was studied extensively. It appeared to be evolving rapidly (Rowe et al. 2002; Allf et al. 2021), exhibiting variation among populations (Atkins et al. 2022) and even individuals (Gibert et al. 2022; da Cunha et al. 2023). Like other behaviours of cold-blooded vertebrates, it is highly dependent on the body temperature (Whitford et al. 2020). On an evolutionary scale, the tail rattling behaviour much preceded the emergence of the rattle (Allf et al. 2016). Most importantly, the rattling fulfils its biological function: its frequency modulation successfully manipulates distance perception of a receiver (Forsthofer et al. 2021) and target species are able to interpret it properly as a deterring signal (but see Caine et al. 2020). The pattern of snake defensive behaviour was recently recognized as a crucial factor determining probability of snakebite and human fatalities (Alves-Nunes et al. 2024).

Notably, the defensive display affects the signal’s recipients. The threatening posture may elevate the saliency of the snake stimulus. Behavioural responses of captive rhesus macaques (*Macaca mulatta*) were stronger to a viper-like snake model in striking than in a coiled posture and also stronger to a snake model in a coiled posture than to an extended sinusoidal snake model (Etting and Isbell 2014). In a neurophysiological experiment, pulvinar neurons of Japanese macaques (*Macaca fuscata*) responded more intensely to images of snakes in open-mouth threat display compared to those depicting snakes in non-threat postures (Van Le et al. 2014). Similarly, children (3–4 years old) detected snakes depicted in a “striking” position earlier than those in a resting one (Masataka et al. 2010). Neither of these two studies considered the taxonomic identity of the examined snake stimuli, but they included viper-like snakes (e.g. pit vipers) as threatening stimuli.

Humans have evolved in the African continent, where they have been exposed to venomous snakes of multiple lineages (for a review see Frynta et al. 2023b), two of which are both deadly venomous and common. These are viperids (Viperidae) and elapids (Elapidae). Viperids are venomous snakes with solenoglyfous fangs. They diverged during the Oligocene (Šmíd and Tolley 2019), and since this period they have been continuously present on the African continent (Frynta et al. 2023b). Elapids are front-fanged (proteroglyphous, Palci et al. 2023) venomous snakes belonging to an Afro-Asian superfamily Elapoidea (Zaher et al. 2019) that appeared in the Early Eocene (about 55 Mya). Elapids started to diversify in the Oligocene (about 30 Mya, Das et al. 2023, 2024). Although elapids are primarily of Asian origin, fossil records suggest that they appeared on the African continent as soon as in the Late Oligocene (about 25 Mya, period of Tanzania, McCartney et al. 2014). The molecular evidence placed the origin of cobras to the lowest Miocene (Zaher et al. 2020). They further diversified into several genera of cobras, king cobras, mambas and African coral snakes. This process is associated mostly with the African continent, although there are Asian king cobras of the genus *Ophiophagus* (Shankar et al. 2021) putatively having also African roots. Typical cobras of the genus *Naja* were reported repeatedly from Miocene localities in Africa (Zaher et al. 2020) and also Europe (Szyndlar and Rage 1990; Syromyatnikova et al. 2021; Ivanov et al. 2022). One lineage of the genus *Naja* then migrated into South Asia and radiated there. In Europe, the cobras persisted until the upper Pliocene period (Villa et al. 2024).

**Fig. 1:**
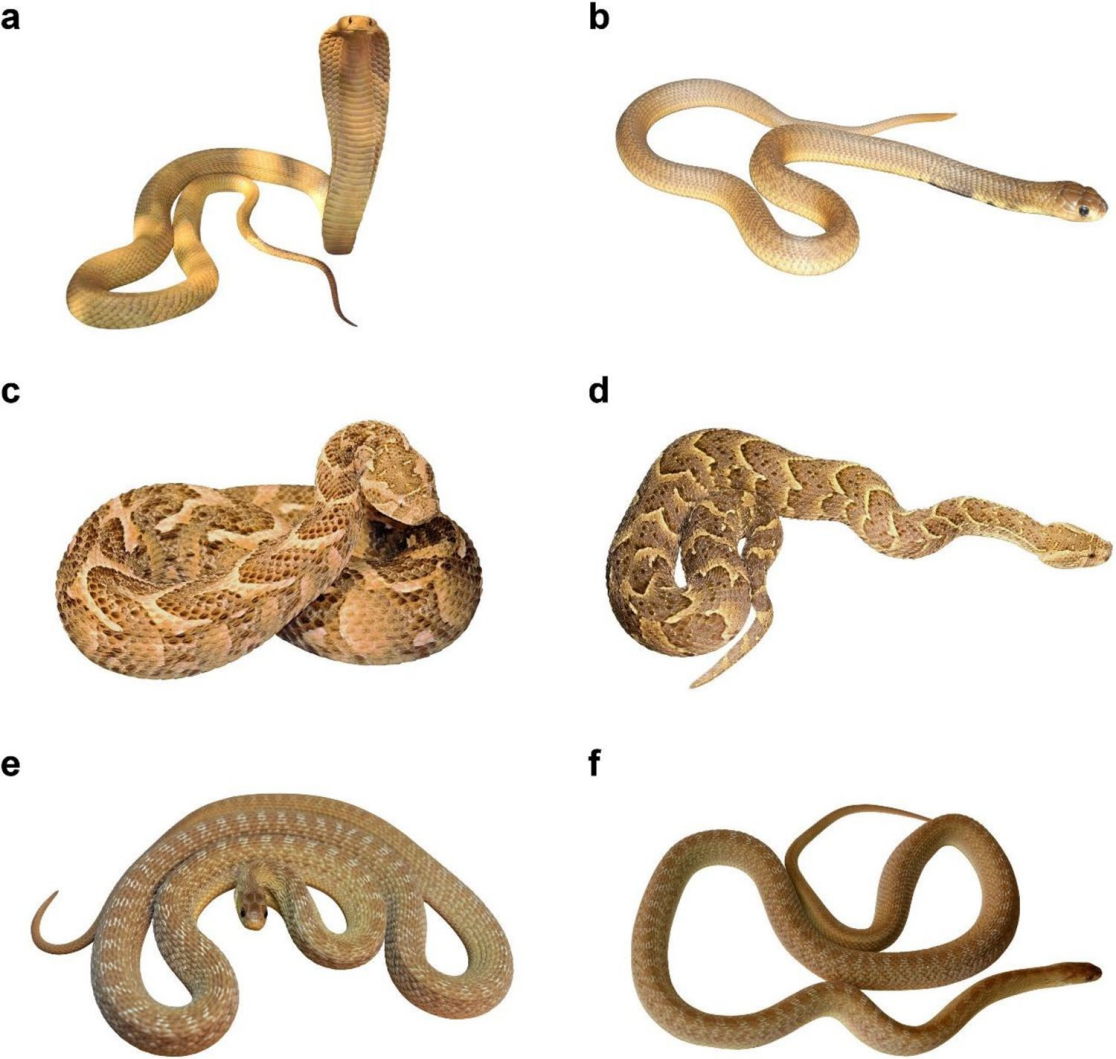
Example of standardized snake stimuli in threatening and resting positions. Typical threatening position of a cobra with hooding (**a**) and resting position (**b**) for the species *Naja nivea*, both positions for vipers (**c**, **d**; species *Bitis arietans*) and non-dangerous snakes (**e**, **f**; species *Dasypeltis gansi*). Original image (**a**) by Willem Van Zyl (CC BY-NC 4.0) and other images (**b**, **c**, **d**, **e** and **f**) by Daniel Frynta and Petra Frýdlová. Adapted into a standardized form by Markéta Janovcová

Cobras are able to flare out the skin and ribs of the elevated neck to enlarge the contours of the body (for mechanisms see Young and Kardong 2010). This hooding posture (Fig. 1a) is displayed to intimidate and warn a predator. Hooding was reported in all species of cobras except nocturnal *Walterinesia*, i.e. in those belonging to the genera *Ophiophagus*, *Pseudonaja*, *Naja,* and *Hemachatus*, while in African coral snakes of the genus *Aspidelaps* it is developed just partially. Phylogenetic reconstructions of the ancestral states suggested that hooding evolved once in the nearest common ancestor of the entire cobra clade and was lost in *Walterinesia* which is strictly nocturnal and thus cannot benefit from optical signalling (Panagides et al. 2017, for phylogenetic trees, see Plettenberg Laing 2019 and Zaher et al. 2020)*.* Moreover, hooding was reported also in African mambas of the *Dendroaspis* branching within the clade of cobras, likely representing a sister group to Asian king cobras of the genus *Ophiophagus.*

The evolution of hooding behaviour in cobras was followed by the acquisition of morphological adaptations to hooding, e.g., neck ribs extension and the appearance of specific patterns on the hood (Jones 2017). Hooding is also associated with increased cytotoxicity (Panagides et al. 2017) and fibrinogenolytic activity of the venom (Bittenbinder et al. 2019). These toxic effects do not contribute to prey immobilization but make the venom more potent to wound the bitten disturber. This further supports the view that hooding has a defensive anti-predator function. Hooding was occasionally reported also outside of the clade of cobras (e.g. in other elapids, Brown et al. 2022), usually in species imitating sympatric cobras (e.g. *Malpolon moilensis*, Psammophiidae). Nevertheless, the case of South American colubrids of the subfamily Dipsadinae (e.g. *Phylodryas*, Jara and Pincheira-Donoso 2015; *Hydrodynastes*, Williams et al. 2022; *Erythrolamprus*, de Alcântara Menezes et al. 2015; *Oxyrhopus*, Dipsadidae, de Assis et al. 2020) suggests that hooding may successfully evolve out of the geographic range of cobras.

Spitting cobras further improved their defensive hooding by spraying the venom. Compared to biting, spitting provides an opportunity to envenom the predator from a safe distance. Spitting cobras are able to track the target (Westhoff et al. 2010) and adjust the distribution according to its distance (Berthé et al. 2009). They are not directly targeting eyes, but the entire face of the disturber (Westhoff et al. 2005; Berthé et al. 2013). Venom spitting evolved independently three times; it was reported in the African rinkhals of the genus *Hemachatus*, in a clade of African spitting cobras (e.g. *Naja ashei*, *N. mosambica*, *N. nigricollis*, *N. nubiae*, and *N. pallida*) and also in some Asian cobras (e.g. *N. sumatrana*, *N. siamensis* and *N. philippinensis*; also in certain populations of *N. atra* and *N. kaouthia*, Paterna 2019; Giri et al. 2021). Evolution of spitting in all these lineages of cobras was associated with an upregulation of phospholipase A_2_ (PLA_2_) toxins, which potentiate the action of preexisting venom cytotoxins to activate mammalian sensory neurons and cause enhanced pain (Kazandjian et al. 2021). It is still uncertain, the pressure of which predator triggered the repeated evolution of spitting in cobras. Kazandjian et al. (2021) suggested that ancient hominins are a plausible candidate. The main argument was the coincidence between the emergence of spitting in African lineage of cobras with the human-chimpanzee split and the emergence of spitting in Asian lineage of spitting cobras with the *Homo erectus* colonization of Asia. Nevertheless, small carnivores like mongooses (Herpestidae) should be also considered (Madsen and Wüster 2022).

In our previous eye-tracking study, we compared visual attention paid to snake stimuli depicted in threatening and resting postures. Pairs of corresponding stimuli were presented on a screen. A presentation involved 20 snake species including highly venomous vipers and elapids. Both fixation number and dwell-time revealed an attentional bias towards threatening stimuli, but while the effect was strong in the case of elapids, it was much smaller or even absent in viperids and other snakes (Štolhoferová et al. 2023). This is significant in the context of the finding that both African and European respondents placed vipers as the top fear evoking snakes, while they placed some deadly venomous elapids near the bottom (Frynta et al. 2023b). It may indicate that while vipers are already protected by their appearance even when resting, defensive behaviour should be essential for the elapids to be recognized by humans as dangerous snakes. Nevertheless, the effects of a threatening position on subjective fear have remained nearly unstudied, until now. The exception represents a cross-cultural study evaluating subjective fear evoked by 37 snake pictures including the Egyptian cobra (*Naja haje*) in both threatening and resting postures where the hooding cobra was rated as top fear evoking in sharp contrast to the resting one (Landová et al. 2018).

In this paper, we compare subjective fear evoked by snakes depicted in threat posture compared to those in resting posture. Moreover, we resorted to a cross-cultural comparison as a powerful tool to support or reject hypotheses of the evolutionary-based origin of specific phenomena. In this case, a cross-cultural consensus would support the innate predisposition for discriminating between snake threat and resting postures while disagreement would instead suggest the role of personal experience or local culture. As in previous papers (Rudolfová et al. 2022; Frynta et al. 2023a, b; Štolhoferová et al. 2023), our respondents were (1) Somali people from the African Horn living in the ancestral environment of arid savannah inhabited by deadly venomous snakes as vipers, mambas and cobras; and (2) Czech citizens.

## Material and methods

###  Respondents

We performed the research at the campus of Amoud University in Borama. The Somali respondents were students (mostly undergraduates) who agreed to voluntarily participate in the experiment. A total of 124 Somali respondents finished the task (for the data see Supplementary Table S1 in Online resource 1). They were 93 men and 31 women. The mean age was 22.12 years (median = 21, range 19–27).

We gathered the Czech respondents among university students, preferentially among those studying technical and other non-zoological disciplines in the capital city of Prague, Czech Republic. They were 95 men and 51 women. The mean age was 20.57 years (median = 20, range 18–36).

###  Stimuli and experimental task

The experimental stimuli were 40 photos of 20 snake species (see Supplementary Table S2 in Online resource 1). We used the same stimuli as in Štolhoferová et al. (2023). We focused on venomous snakes represented by 16 species in our set. According to their fang morphology, body shape (morphotype), behaviour and phylogeny, we distinguished two distinct categories, further referred to as the “vipers” and “elapids”. The vipers were represented by eight species of the family Viperidae (*Bitis arietans*, *B. rhinoceros*, *Cerastes cerastes*, *C. vipera, Echis pyramidum*, *E. sochureki*, *Montivipera raddei* and *Vipera ammodytes*). The “elapids” were represented by 5 cobras (*Naja atra*, *N. haje*, *N. melanoleuca*, *N. naja* and *N. nivea*), a black mamba (*Dendroaspis polylepis*) and an African coral snake (*Aspidelaps lubricus*), all belonging to a single monophyletic clade within the family Elapidae. We included boomslang (*Dispholidus typus*) in the category of “elapids” despite its phylogenetic placement within the family Colubridae. It is a deadly venomous snake resembling mambas by the morphology of the fangs and cranium, slender body and threatening posture characterized by elevating head and neck (Westeen et al. 2020). In addition, we included two African pythons (*Python sebae* and *P. regius*) of the family Pythonidae, and two species belonging to the family Colubridae: the egg-eater *Dasypeltis gansi* and the Arabian cat-snake *Telescopus dhara*. These are further referred to as “non-venomous”.

Each species was represented by two photographs, one depicting the snake in a relaxed (resting) posture, and one taken when the animal was in alerted threatening posture. When available, pictures of the same snake individual were used. Most photos of the stimuli came from authors’ personal archives (for a complete list of stimuli sources, see Supplementary Table S2 in Online resource 1). As in previous studies (e.g. Frynta et al. 2023a, b), we digitally placed the snakes on a white background and resized them so that the pictured stimuli were of a similar size. We performed this adjustment to avoid possible effects of the background and size of the stimulus on rankings. Then, we printed the final stimuli as photographs 100 × 150 mm in size. We previously showed that fear evaluation of standardized pictures correlates with that of live animals at least in terms of ranking order (Landová et al. 2012) which was the method utilized in this study. For the example of experimental stimuli, see Fig. 1.

We applied a rank-order method and followed the same testing procedure as in our previous studies performed in Somaliland (Frynta et al. 2023a, b). At the beginning of the task, a respondent was standing in front of a well-lit table. We provided him/her with a set of 40 pictures packed in random order. We asked the respondents to imagine the pictures as real animals. Then, we asked him/her to place all stimuli on the table in a random assemblage. This sometimes required assistance to ensure that the stimuli were oriented properly, i.e. the top margins of the stimuli were oriented towards the top of the table. The task was to pick up the picture of an animal that was the most fear-evoking and then to pick up the second most fear-evoking one, until he/she picked up the least fear-evoking stimulus on the table. In the end, the respondent had a whole pack of pictures in his/her hand. Finally, each respondent was asked for age information and their gender was recorded. The entire task took most respondents approximately 10 to 15 min. The picture order in the pack was then coded from 1 (the most fearful one) to 40 (the least fearful one), further referred to as ranks. We repeatedly demonstrated that mean ranks were highly correlated to scores produced by the 5- or 7-point Likert scale (e.g. Frynta et al. 2010; Rádlová et al. 2019).

###  Data analysis

Instead of median ranks, we adopted the following index to obtain more intuitive values increasing with fear (not decreasing as original ranks and its means) and ranging from 0 to 100: Fear = 100 − (100 × (median rank − 1) / (the number of examined stimuli − 1)). Nonetheless, since the primary data were ranks, we still took advantage of non-parametric statistical methods (if possible) which are specifically suited for ordinal variables. (1) To quantify agreement among the respondents, we computed Kendall’s coefficient *W*, as implemented in the package irr (Gamer et al. 2022). (2) To assess the variance in the original data which is constrained by country, gender, age and country-gender interaction, we employed redundancy analysis (RDA) with a permutation test, as implemented in the package vegan (Oksanen et al. 2020). (3) To compare the fear ranking of the same stimulus presented in threatening and relaxed positions, we employed a sign-test. Since we a priori planned to perform the tests for each combination of 20 species and nationality (Somali versus Czech), we applied Bonferroni correction for 40 comparisons. (4) To compare the mean ranks of individual stimuli, we first calculated the Friedman test enabling us to prove that the effect of species on rank is significant. Then, we employed the post hoc Friedman-Neményi test permitting reliable multiple comparisons among the stimuli. These tests were performed using PMCME and PMCMRplus packages (Pohlert 2014). All these calculations we carried out in the R-environment (R Development Core Team 2022).

In addition, we calculated the difference between the mean fear of the threatening and resting stimulus for each of 20 snake species (separately for Somali and Czech samples) and correlated them with the corresponding differences in mean dwell time recorded for the same pairs of stimuli by Štolhoferová et al. (2023). This allowed us to compare mean attentional bias towards snakes in threatening postures and mean subjective fear elicited by the same stimuli. Please take notice that this was not an individual-level comparison, since participants involved in the present and the previous study did not overlap.

All data associated with this study are provided in Supplementary Table S1, the descriptive statistics in Supplementary Table S3 in Online resource 1.

## Results

###  The agreement among respondents

We found significant agreement among the 124 Somali respondents as well as among the 146 Czech ones. Kendall’s coefficients of concordance (Wt) were 0.298 (*χ*^2^_(39)_ = 1442, *P* <  < 0.0001) and 0.263 (*χ*^2^_(39)_ = 1496, *P* <  < 0.0001), respectively. Thus, it is meaningful to calculate means and compare the subjective fear among examined stimuli. Agreement calculated for the pooled data was also significant (Wt = 0.244, *χ*^2^_(39)_ = 2568, *P* <  < 0.0001).

###  The effect of respondents’ characteristics and data partitioning

We assessed the effects of nationality, gender, age, and nationality-gender interaction on the ranking of the stimuli. The best RDA model (*AIC* = 2243.4) included only one factor, nationality (Somali vs Czech), constraining 4.66% of the variation in the entire data set (ANOVA: *F*_(1,268)_ = 13.09, *P* < 0.001). The permutation test revealed that the effects of gender, age, and nationality-gender interaction on the ranking of the stimuli are negligible (*P* > 0.05; see Supplementary Table S4 for full results). Thus, we further analysed the Somali and Czech data separately.

###  The effect of threatening posture

**Fig. 2:**
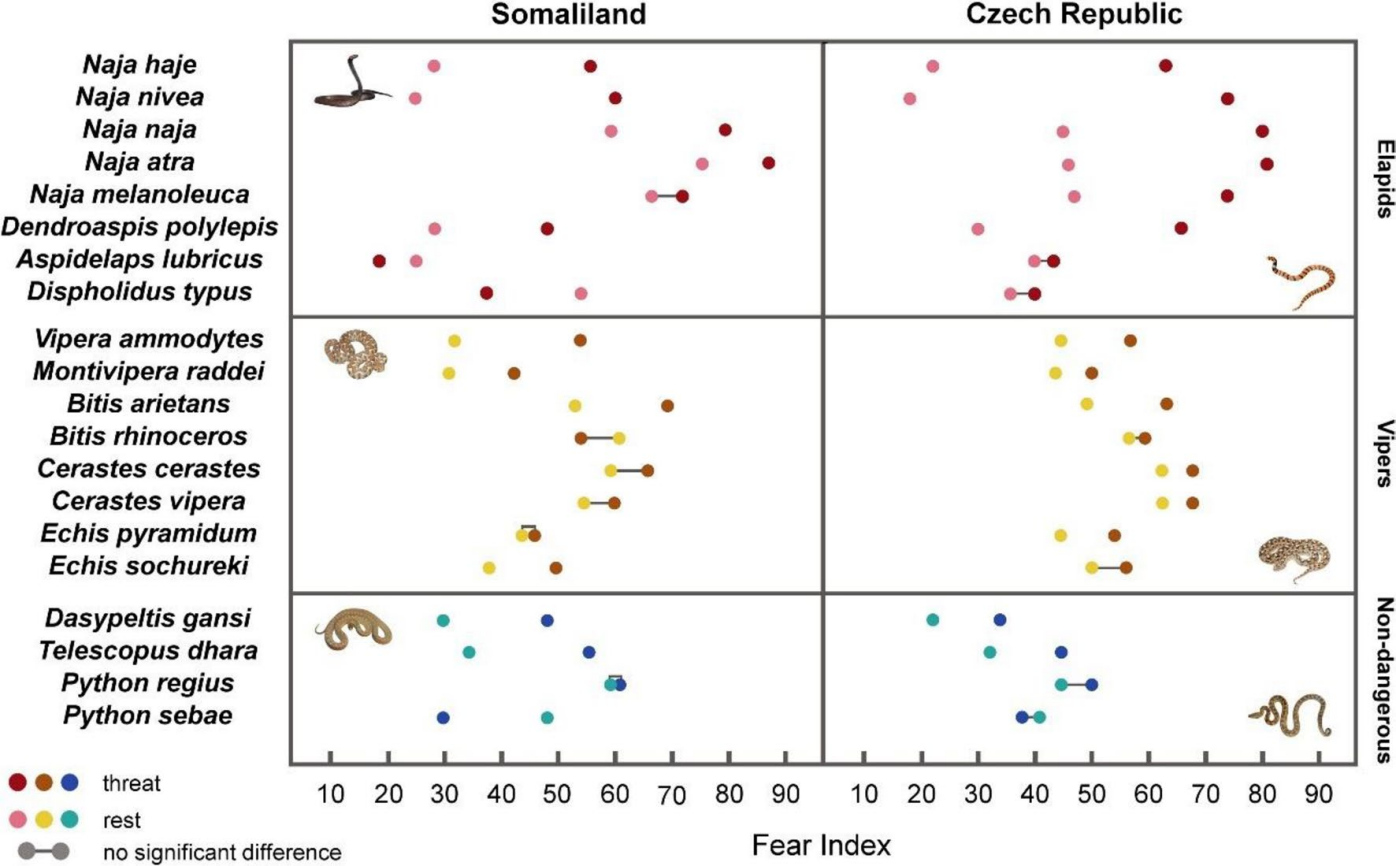
Comparison of fear ratings elicited by threatening and resting postures of snakes in the two tested nationalities. The Fear Index shows the level of fear of each stimulus, the higher the number, the more fear the image evokes in the humans. For each species, the darker colour (red, brown and blue) shows the rating of the threatening posture, and the lighter colour (pink, yellow and turquoise) shows the rating of the resting posture. In case no significant difference in ratings was found between the positions, the two points are connected by a straight line

We compared the subjective fear elicited by stimuli depicting threatening and resting snakes of the same species (Fig. 2 and Supplementary Table S5 in Online resource 1). In the Somali dataset, 16 of 20 species elicited on average more fear when presented in a threatening posture (sign test: *Z* = 2.46, *P* = 0.0139) and 11 of these partial comparisons were significant (Bonferroni corrected sign-test). These included 5 elapids (cobras *N. haje*, *N. nivea*, *N. naja*, *N. atra,* and black mamba *D. polylepis*), 4 viperids (*V. ammodytes*, *M. raddei*, *B. arietans,* and *E. sochureki*) and two colubrids (*D. gansi* and *T. dhara*). The inverse relationship (i.e. resting posture evoking more fear than the threat) was significant in 3 cases; all concerning species in which the threatening position was less pronounced or untypical (African coral snake *A. lubricus*, boomslang *D. typus,* and African rock python *P. sebae*).

In the Czech dataset, all species except *P. sebae* (19 of 20) elicited on average more fear when presented in a threatening posture (sign test: *Z* = 3.80, *P* < 0.0002), and 14 of these comparisons were significant. These were 6 elapids (all examined cobras of the genus *Naja* and black mamba *D. polylepis),* 6 viperids (all except of *B. rhinoceros* and *E. sochureki*) and both non-venomous colubrids (*D. gansi* and *T. dhara*).

The relationship between the mean fear evoked by the snake (*n* = 20 species) in the threatening posture with that in the resting position was significant in the Somali dataset (*r*_Spearman_ = 0.558, *t* = 2.85, *P* = 0.0106), but not in the Czech one (*r*_Spearman_ = 0.402, *t* = 1.86, *P* = 0.0793).

###  Patterns of elicited fear among the stimuli

Friedman test revealed that the effect of the stimulus on perceived fear was highly significant in both Somali and Czech datasets (*P* <  < 0.0001). This enabled us to calculate Friedman-Neményi comparisons among the 40 examined stimuli (20 snake species, each depicted in both threatening and resting positions). Out of 780 post hoc comparisons among the stimuli, 423 (53.8%) and 420 (54.2%) were significant in the Somali and the Czech datasets, respectively (*P* < 0.05, for the matrices of *P* values, see Supplementary Tables S6 and S7 in Online resource 1).

In the Somali dataset, cobras of the genus *Naja* in threatening posture were salient stimuli occupying the 1st (*N. atra*), 2nd (*N. naja*), 4th (*N. melanoleuca*), 10th (*N. nivea*), and 15th positions (*N. haje*). The first three of these species occupied top positions even when presented in the resting posture (3rd *N. atra*, 6th *N. melanoleuca*, 13th *N. naja*), while resting *N. haje* and *N. nivea* belong to the stimuli evoking very low fear (37th and 39th, respectively). Low fear elicited also the black mamba (*D. polylepis*: 25th and 36th position for threatening and resting, respectively), African coral snake (*A. lubricus*: 40th and 38th) and boomslang (*D. typus*: 30th and 18th). Eight of 16 viper stimuli elicited fear above the median value.

In the Czech dataset, the saliency of threatening elapids is even more apparent. The cobras and mamba depicted in this posture occupied the first four (1st *N. atra*, 2nd *N. naja*, 3rd *N. melanoleuca,* and 4th *N. nivea*), 7th (*D. polylepis*) and 9th (*N. haje*) positions. Threatening vipers (*C. vipera*, *C. cerastes,* and *B. arietans*) are on the 5th, 6th and 8th positions, respectively. The remaining positions above the median fear are occupied by 10 vipers and a python (19th *P. regius* in threatening posture).

###  The cross-cultural agreement

**Fig. 3:**
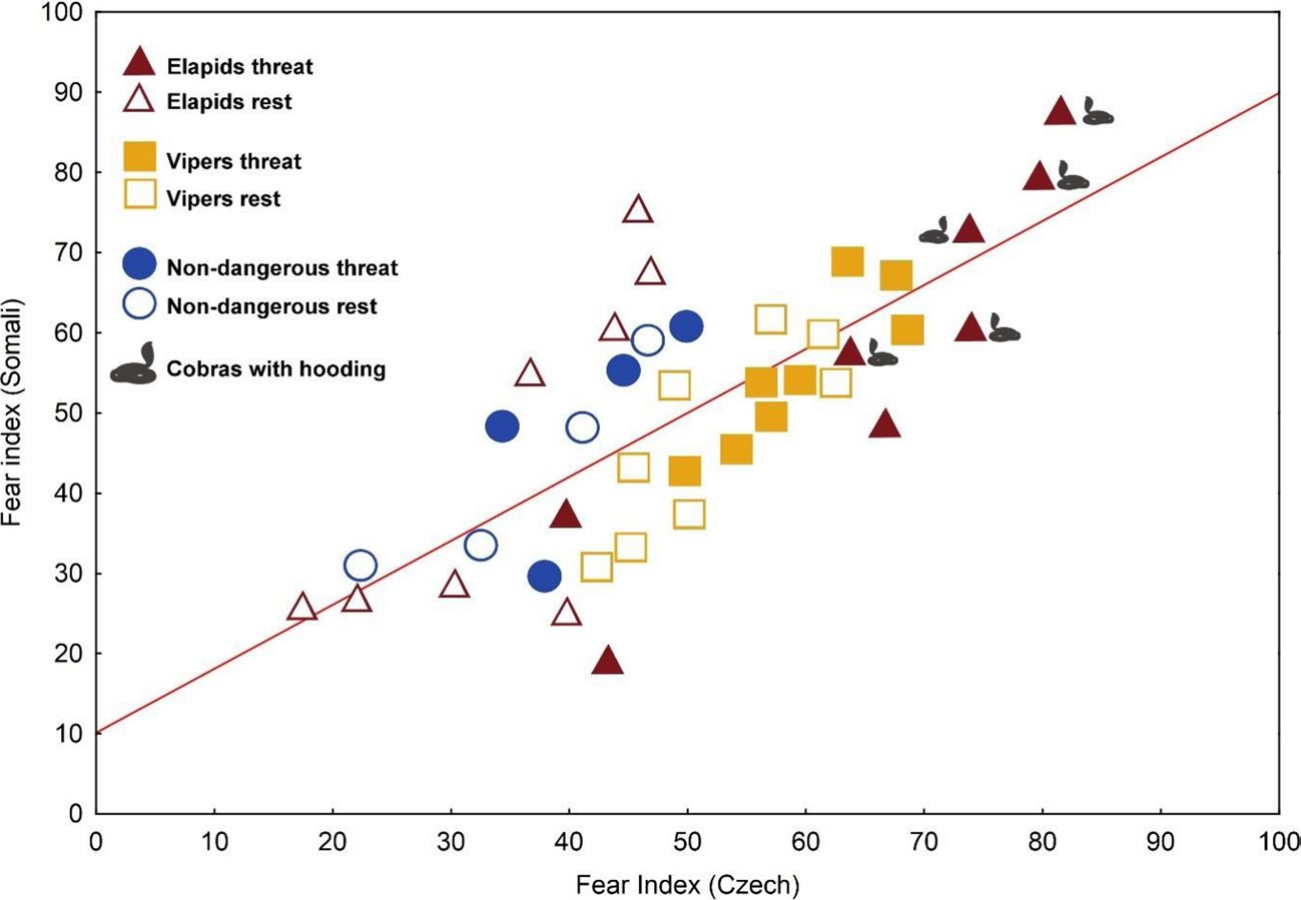
Cross-cultural agreement in the evaluation of different snake positions according to fear. The solid symbols represent the threatening position of elapids and boomslang (triangle), vipers (square) and non-dangerous snakes (circle). Empty symbols indicate these categories of snakes in a resting position. The grey snake symbol indicates cobras of the genus *Naja *exhibiting fully expressed hooding. The Spearman correlation coefficient for snake ratings of Somali and Czech respondents was 0.739 (*p* < 0.0001)

Subjective fear elicited by the stimuli in Somali and Czech respondents was closely correlated (Fig. 3). The Spearman correlation coefficient for the entire set of the 40 stimuli (threatening and resting positions combined) was 0.739 (*t*_(*n* −2)_ = 6.76, *P* < 0.0001). The strength of this association was comparable when calculated separately for threatening (0.788, *t*_(*n* −2)_ = 5.43, *P* < 0.0001) and resting positions (0.740, *t*_(*n* −2)_ = 4.67, *P* = 0.0002).

### The between-stimuli correlation of attentional bias and relative fear saliency

The correlations between the differences within pairs of the threatening and resting stimuli (*n* = 20) in the subjective fear and in the mean dwell time were significant both in the Somali (*r*_Spearman_ = 0.620, *t* = 3.35, *P* = 0.0036) and the Czech (*r*_Spearman_ = 0.445, *t* = 2.11, *P* = 0.0492) samples.

## Discussion

We showed that a snake depicted in a threatening posture typically evokes more subjective fear than the same snake depicted in a resting posture no matter the examined snake morphotype (lineage). Moreover, we found only a weak correlation between the subjective fear of the same snakes presented in threatening and resting postures. This suggests that humans can interpret warning signals of snakes which may in turn be used to reliably predict the probability of subsequent strikes (Glaudas and Winne 2007). Nevertheless, the effect of posture was much more pronounced in the case of certain elapids, specifically in the true cobras of the genus *Naja* and partially also in the black mamba. This finding is in accord with our previous eye-tracking study demonstrating a strong attentional bias favouring threatening elapids over the resting ones (Štolhoferová et al. 2023). We showed that the attentional bias extracted for each pair of threatening and resting stimulus is a significant predictor of corresponding disparity in the elicited subjective fear. Even though this relationship was fairly tight in the Somalis (*r*_Spearman_ = 0.620), the correlation was noticeably weaker in the Czechs (*r*_Spearman_ = 0.445), and for both datasets, the effect of other factor(s) is very likely. Hence, subjective fear is not driven solely by attention, and conversely, attention is not solely driven by subjective fear. Subjective fear might be also driven by, for example, categorization, superficial similarity to the prototypic stimuli, or presence of specific features generally associated with danger (e.g. bared teeth). Visual attention might be, on the other hand, driven by primary features of the stimuli (e.g. complexity), high arousal (of both positive and negative valence) or ambiguity. This finding is especially important for studies on attention designed on a finer scale in which most stimuli are fear-eliciting, and the aim is to compare relative attention dedicated to each stimulus type.

###  Cross-cultural agreement

In our previous paper, we showed that subjective fear is associated with certain morphological categories of snakes, rather than certain species (Frynta et al. 2023b). This was also the case for this study. We failed to support the hypothesis that Somalis exhibit selective fear of principal deadly venomous snakes inhabiting their country. The stimuli depicting dangerous and native species of Northeast African carpet viper (*E. pyramidum*), black mamba (*D. polylepis*), and boomslang (*D. typus*) induced fear well below the median value.

Somalis and Czechs are exposed to sharply contrasting experiences with snakes. In Somaliland, we frequently meet survivors of snakebites and envenoming (see also snakebite statistics and reports from the neighbouring countries of the African Horn, e.g., Aga et al. 2014 and Fekadu 2016). Contrary, the risk of a snakebite is negligible in Central Europe (Valenta 2010; Paolino et al. 2020). Moreover, elapid snakes like cobras have been entirely absent in the European faunas since their final extinction in the late Pliocene, i.e. much before the first representatives of the genus *Homo* entered Eurasia. Therefore, any experience of Czech respondents with elapid snakes relies solely on zoos, media and culture. Thus, one could expect considerable differences in the ranking of snake stimuli by the Somali and Czech respondents.

In spite of this, we detected a considerable agreement between the ranking of stimuli by Somali and Czech respondents. Multivariate analysis showed that the effect of country constrained just less than 5% of the stimuli ranking. Moreover, the correlation between the mean ranks of the individual stimuli calculated from Somali and Czech datasets was high (*r*_Spearman_ = 0.739; Fig. 3) and comparable to those reported in similar studies (Landová et al. 2018; Frynta et al. 2023b). It remained nearly unchanged when calculated separately for stimuli in resting and threatening posture. Thus, the great between-species variability in the threat postures has little effect on cross-cultural agreement.

###  The threatening posture of cobras

The pronounced subjective fear of hooding cobras identified in this study can be interpreted as a consequence of the evolution of cobras which have developed an anti-predatory strategy that effectively deters mammalian predators, including humans. One possible scenario is that evolutionary experience with cobras has specifically altered the primate brain to recognise a cobra’s threatening stance. There is clear evidence that the selective pressure exerted by cobras on our primate ancestors was strong enough to alter their biological characteristics. Harris et al. (2021) mapped the degree of resistance to elapid α-neurotoxins onto the phylogenetic tree of primates and found that lineages that have coexisted with cobras for an extended period have developed increased resistance to their venom. These include Old World monkeys (Cercopithecoidea) and apes (Hominoidea). Interestingly, the most resistant are chimpanzees, gorillas, and humans. This may suggest that human evolution in the African continent was significantly affected by the occurrence of cobras and/or mambas (Harris et al. 2021).

Nevertheless, one can also argue for a simpler evolutionary scenario. The cobras’ elevated/hooding posture might only exploit pre-existing cognitive mechanisms that interpret an enlarged body outline, an upright posture (Prokop et al. 2021; Coss 2020) or patterning as cues of potential dangerousness. These cues are almost universally utilized by animals in both inter- and intra-species interactions and can be even traced in many human cultural artefacts. Thus, elapids adopting an upright posture as a defensive threat display may have been a highly effective strategy, as it would have required little to no evolutionary time for predators to recognize and properly respond to it. Nonetheless, we can reasonably speculate that the strong fear response to deadly venomous elapids has been further enhanced by the continuous evolutionary experience with these snakes in Africa. Both of the mutually non-exclusive scenarios are in line with Isbell’s hypothesis that repeated encounters with venomous snakes have shaped primate brains (Isbell 2006, 2009).

## Conclusions

We convincingly showed that snakes in a defensive threat posture evoked greater fear than the same species in a relaxed resting posture. This result held true for both Somalis and Czechs who ranked the species very similarly, in general. The current study therefore adds to the substantial amount of evidence that snakes are perceived very similarly across many very distant and unrelated cultures. However, not all kinds of defensive postures were equally efficient in evoking fear in humans. Some species exhibiting salient defensive posture such as hooding cobras may increase the subjective fear they evoke substantially. On the other hand, vipers in resting posture were already perceived as highly fear-eliciting hence display of threats could have elevated the fear only relatively little (the so-called ceiling effect). We recommend other researchers to pay attention to this interaction between investigated species and their body posture in their future study designs.

In our previous study (Frynta et al. 2023b), we reported that people, even those living in Africa, exhibit only limited fear of cobras and mambas when they evaluate the visual stimuli in resting posture. Considering the cobras’ venomous nature, this finding was peculiar. In this study, we show that humans can avoid highly risky interactions with snakes by responding to snake threat displays with elevated fear and subsequent fear response. Even though fear elicited by all types of snakes in threatening posture increases, cobras surpass other snakes in their ability to evoke human fear through their specific threat display—hooding. In fact, elapids in a threat posture can even get ahead of vipers, which are typically considered the most fear-inducing snakes. This can be primarily attributed to the behavioural evolution of cobras, which may have tapped into pre-existing cognitive mechanisms in mammals, as well as the prolonged co-existence of cobras and humans in Africa, likely intensifying the fear response to cobra threats even further.

## Supplementary Information

Below is the link to the electronic supplementary material.

Supplementary file1 (XLSX 120 KB)

## Data Availability

All the data generated or analysed during this study are included in this published article in its Supplementary Information files (Online resource 1).
